# Giant Cushioning Effect in Facile Polymer/Nanoclay-Coated Flexible Polyurethane Foams

**DOI:** 10.1021/acsapm.4c01437

**Published:** 2024-08-22

**Authors:** Wenfei Ji, Qicheng Zhang, Jeroen S. van Duijneveldt, Wuge H. Briscoe, Fabrizio Scarpa

**Affiliations:** † School of Chemistry, University of Bristol, Cantock’s Close, Bristol BS8 1TS, U.K.; ‡ Bristol Composites Institute, University of Bristol, University Walk, Bristol BS8 1TR, U.K.

**Keywords:** polyurethane foam, sepiolite, energy dissipation, damping, mechanical properties

## Abstract

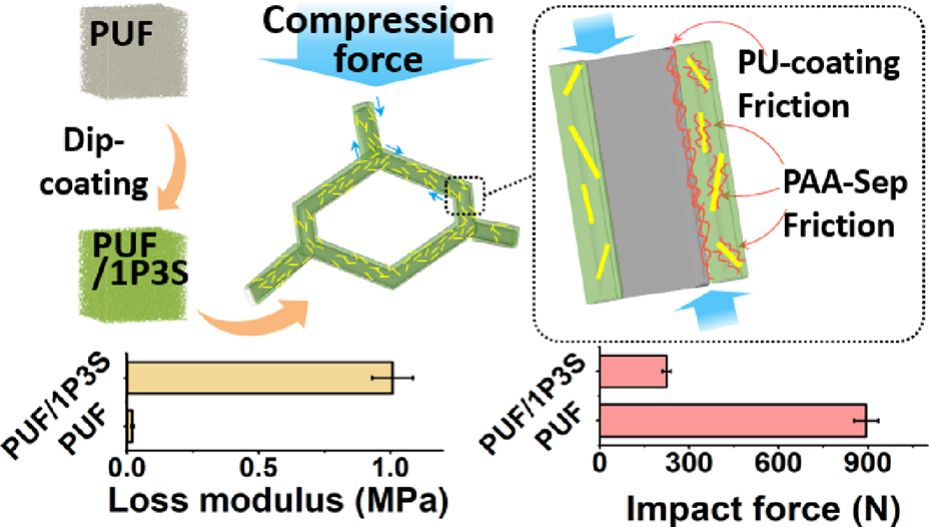

In this work, a flexible polyurethane (PU) foam/polymer/clay (PUF/PAASep) composite is prepared via a simple dip-coating method. The composite exhibits excellent damping properties under quasi-static compression, vibration transmissibility, and impact resistance. For the composite preparation, sepiolite (Sep) dispersion in a polyacrylic acid (PAA) solution is first homogenized and evaluated using microscopy, and the obtained PAASep suspension is used to coat the PU foam uniformly for optimization of the quasi-static mechanical performance of the foam composites. The PU foam struts coated with 1 wt % PAA and 3 wt % sepiolite are strengthened, resulting in an 8-fold improvement of the stiffness and three-times increase of the impact force resistance compared to the uncoated PU foams. More importantly, the PU foam composites show a remarkable vibration damping capability, with the loss modulus 57 times that of the uncoated PU foams, enabled by micro friction and stick–slip effects mediated by the PAASep coatings. The facile prepared PAASep-coated PU foams have significant potential for cushioning, packaging, and broad engineering applications involving energy absorption.

## 1 Introduction

As the transport of goods has expanded globally, so has the need for advanced materials with enhanced cushioning effects. Effective cushioning materials are helpful to attenuating vibration and noise during machine operation,^1−3^ preventing unexpected shock and impact in packaging,^4−6^ protecting the body in medical care,^7,8^ maximizing comfort,^9,10^ or even resisting seismic waves^11^ because of the significant energy damping performance.

Damping refers to the loss of kinetic energy by dissipation in a mechanical system. The energy is usually dissipated and transformed into heat via internal friction, deformation, viscous drag, or a combination of those, depending on the nature of the damper. The damping capability of a material is usually described using the complex modulus approach that relates to the storage modulus, damping ratios, loss factors, loss modulus, and dissipated energy.^12−14^ In previous studies, researchers have designed various damping material technologies, such as those based on metals,^15^ alloys,^16^ ceramics,^17^ and polymers like rubbers, epoxy, and nylon composites.^18,19^ However, it is challenging to prepare materials with effective damping that exhibit both high storage moduli and loss factors over extended frequency ranges and/or temperatures.^20^ Materials with a high density such as alloys or ceramics do not provide lightweight solutions and contribute to elevated transportation costs and energy consumption, thus limiting their use for practical damping applications. It is therefore necessary to design damping materials with lightweight characteristics and significant damping capability.

Over the past decades, polymeric foams have been explored as cushion materials.^9,21−25^ A polymeric foam comprises a polymer in a cellular structure with a high-volume fraction of air. Flexible polyurethane foam (PUF), a typical polymeric foam, shows interesting lightweight characteristics, accessibility, and low cost and can undergo large deformations and absorb energy during a deformation–recovery cycle loading.^26,27^ PUFs can be used as impact cushions and vibration pads. The mechanical and multifunctional performance of PUFs has also been tailored by adding one or more materials using a facile coating method. This method can be traced back to 1994, when Yanagi and co-workers developed an artificial trachea using coated PUF as a base material.^28^ In recent years, PU foam composites have been prepared using a variety of coating materials, ranging from carbon,^29,30^ inorganic materials, and electrolytes to polymers, shear-thickening materials, and aerogels^14,31−33^ to enhance stiffness, mechanical damping, and general dielectric properties of the PUFs and broaden the application of conventional foams.^34−36^

Among various coating materials evaluated, the natural abundance, low cost, and high modulus (∼100 GPa) of clay particles make them an attractive choice as nanofillers.^36,37^ In recent years, PU foams/clay composites have been designed and fabricated to improve the mechanical strength, flame resistance, thermal stability, and adsorption capacity of foams.^38−44^ The potential of increasing vibration damping and impact energy has not been fully investigated. On the other hand, clay-coated PUFs have been usually prepared via layer-by-layer (lbl) assembly, taking advantage of the electrostatic attraction between the clay surfaces and other coating materials (i.e., the PUF/chitosan/sepiolite composites in our previous work^36^). The repeated process within a lbl assembly coating is, however, time-consuming, especially when a large amount of clay needs to be coated. An efficient and time-saving method for fabricating PUF/clay composites is, therefore, highly sought after. Polyacrylic acid (PAA) and its sodium salt are known as dispersants for various clay particles due to the stabilizing effect on the clay colloids.^45,46^ It can also potentially mediate adhesion to other polymers, which helps fabricate clay–polymer composites.

In this work, we have developed a one-step coating process using sepiolite clays and poly(acrylic acid) to enhance the mechanical properties of flexible open-cell polyurethane foams. The mixing tools have been optimized to achieve a uniform coating and the best mechanical performance. The stiffness, energy dissipation, and damping capability of the foams with different coating formulations have been tested via quasi-static compression, vibration transmissibility, and impact tests.

## 2 Experimental Section

### 2.1 Materials

Sepiolite nanorods (Sep, Si_12_O_30_Mg_8_(OH)_4_(OH_2_)_4_·8H_2_O with a diameter 20–30 nm and a length ∼300 nm) and poly(acrylic acid) (PAA, molecular weight 250,000 g mol^–1^, 35% in water, and density 1.15 g/mL) were purchased from Sigma-Aldrich. Deionized (DI) water was purified using a Millipore Milli-Q Plus system with a resistivity of 18 MΩ cm. Flexible open-cell polyurethane foams, with an apparent density of 28.7 kg m^–3^, were purchased from SM Foam Ltd. All raw materials were used as received, unless otherwise stated.

### 2.2 Preparation of PUF/PAASep Composites

**Figure 1:**
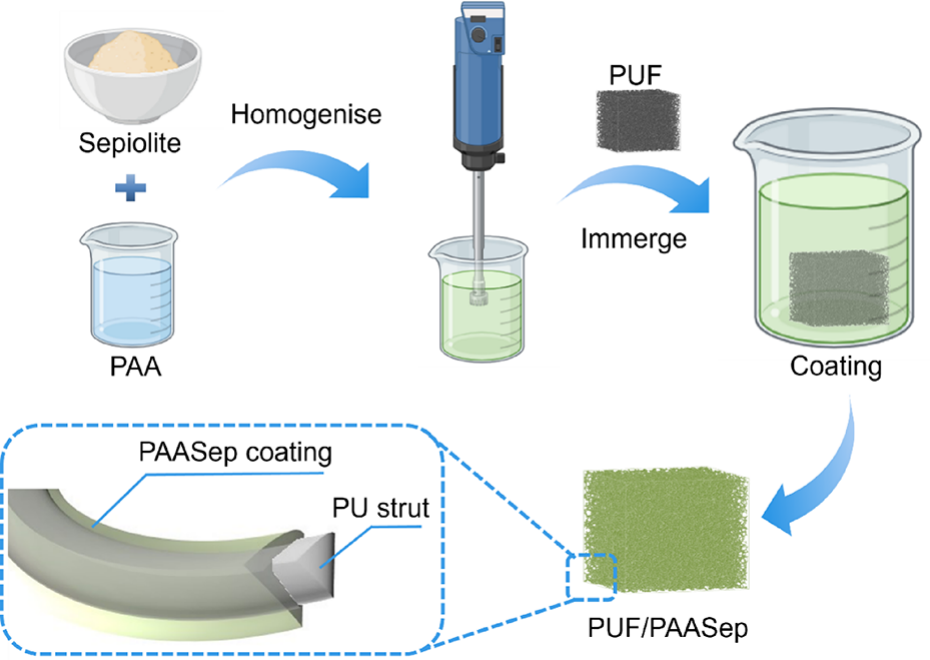
Preparation of PAA and sepiolite-coated polyurethane foams.

The procedure for PAA/Sep coatings is shown in Figure 1. The polyurethane foams were first cut into cuboidal samples of 30 × 30 × 15 mm (with ±1 mm precision) using a hot wire cutter. The foam cubes were labeled using a marker pen and dried at 60 °C for 2 h before coating. The foams were weighed individually, with a mass of 0.38 (±0.02) g. PAA was then diluted in the DI water at a concentration of 1 wt % to make full use of the energy damping potential of PAA (shown in Figure S1 in the Supporting Information). It was then mixed with sepiolite particles at a series of designated concentrations. Four mixing methods were considered for modifying the clay dispersion, to evaluate how the dispersion affected the coating homogeneity and mechanical performances: (1) stirring with a magnetic bar at a rate of 600 rpm (marked as M); (2) an overhead mechanical blade mixer at a rate of 600 rpm (marked as B); an IKA UltraTurrax high shear mixer at a rate of (3) 6000 rpm (marked as UT6) or (4) 10,000 rpm (UT10). Each mixing method was carried out at room temperature for a duration of 10 min. The PUF samples were immediately immersed into the mixed PAA/Sep suspension and squeezed to expel the air inside the foams. The foams were then left immersed for 10 min before being taken out from the suspension and dried at 80 °C for 10 h. No weight loss caused by unevaporated water was observed in the samples, indicating that the foams were dried out. To eliminate the effect from the humidity, the foam samples were then placed in a chamber with a saturated Mg(NO_3_)_2_ solution to keep a constant relative humidity (RH) of 52% for at least 48 h.

Table 1 lists the formula of each foam sample and the used mixing tools.

**Table 1:** Coating Composition and Mixing Condition of the PUF/PAASep Samples

samples	PAA content (wt %)	Sep content (wt %)	mixing tool	rate (rpm)	mass gain (%)	sample density (kg m^–3^)
PUF	-	-	-	-	-	28.8 ± 0.6
PUF/1P	1	-	-	-	27	36.7 ± 0.4
PUF/1P1S	1	1	UltraTurrax	10,000	83	47.1 ± 0.9
PUF/1P2S (M)	1	2	magnet bars	600	122	55.1 ± 0.2
PUF/1P2S (B)	1	2	overhead blade mixer	600	111	50.9 ± 0.5
PUF/1P2S (UT6)	1	2	UltraTurrax	6000	115	52.9 ± 0.4
PUF/1P2S (UT10)	1	2	UltraTurrax	10,000	101	49.2 ± 1.7
PUF/1P3S	1	3	UltraTurrax	10,000	146	60.6 ± 0.6
PUF/1P4S	1	4	UltraTurrax	10,000	198	71.2 ± 0.8
PUF/1P5S	1	5	UltraTurrax	10,000	226	76.7 ± 1.0

### 2.3 Optical Microscopy

The optical images of the PAA/Sep mixture were obtained using a bright-field Olympus BX 51 microscope in differential interference contrast (DIC) mode. To observe the distribution of the clay particles, polarized light microscopy (PLM) was applied. The images were taken using a 10× objective and a Pixelink 5MP color CCD PL-B625CU camera with 2592 × 1944 pixels (1 pixel = 0.43 μm). The PLM images were then processed by using ImageJ to extract the diameter of the sepiolite particles before the particle distribution calculation.

### 2.4 Scanning Electron Microscopy (SEM)

The surface morphology of the PUF samples was observed using a Hitachi TM3030 tabletop scanning electron microscope with a secondary electron detector at an accelerating voltage of 15 kV. The samples were cut into a thickness of 1 mm for observation.

### 2.5 Mechanical Tests

In all mechanical tests, five identical samples with a size of 30 × 30 × 15 mm were evaluated for each group and used to extract the average and standard deviation of the metrics. The calculation of the parameters related to all the mechanical tests is listed in the Supporting Information. The quasi-static compressive tests, vibration and transmissibility tests, and impact tests were carried out using the rigs described previously.^36^

The compressive tests were performed using a universal testing machine (Shimadzu, 1 kN load cell) fixed to double metal plates. The foam samples were given a preload of 0.7 N before undergoing five compressing–releasing cycles at a rate of 1 mm min^–1^ to avoid Mullin’s effect.^47^ The maximum strain was 20%. The Young’s moduli were calculated according to our previous study^36^ as the slopes of the fitted line within the 0–0.5% strain. During the stress relaxation tests, the samples were compressed to 30% strain, then kept under this loading for 5 min.^36^ The dynamic transmissibility tests were performed using a one-dimensional vibration rig.^47^ The samples were fixed and subjected to seismic vibration with a series of top masses to modify the resonance frequency of the system (32.5, 64.0, 107.2, 153.4, and 201.6 g). The impact properties were investigated using a custom small drop tower rig.^48^ The drop tower mass was 0.36 kg, releasing impact energies of 0.2, 0.4, and 0.8 J, corresponding to a drop mass height of 58, 116, and 232 mm, respectively.

## 3 Results and Discussion

### 3.1 Effect of the Mixing Tools on the PUF/1P2S Composites

#### 3.1.1 Clay Distribution in the PAA Solution

**Figure 2:**
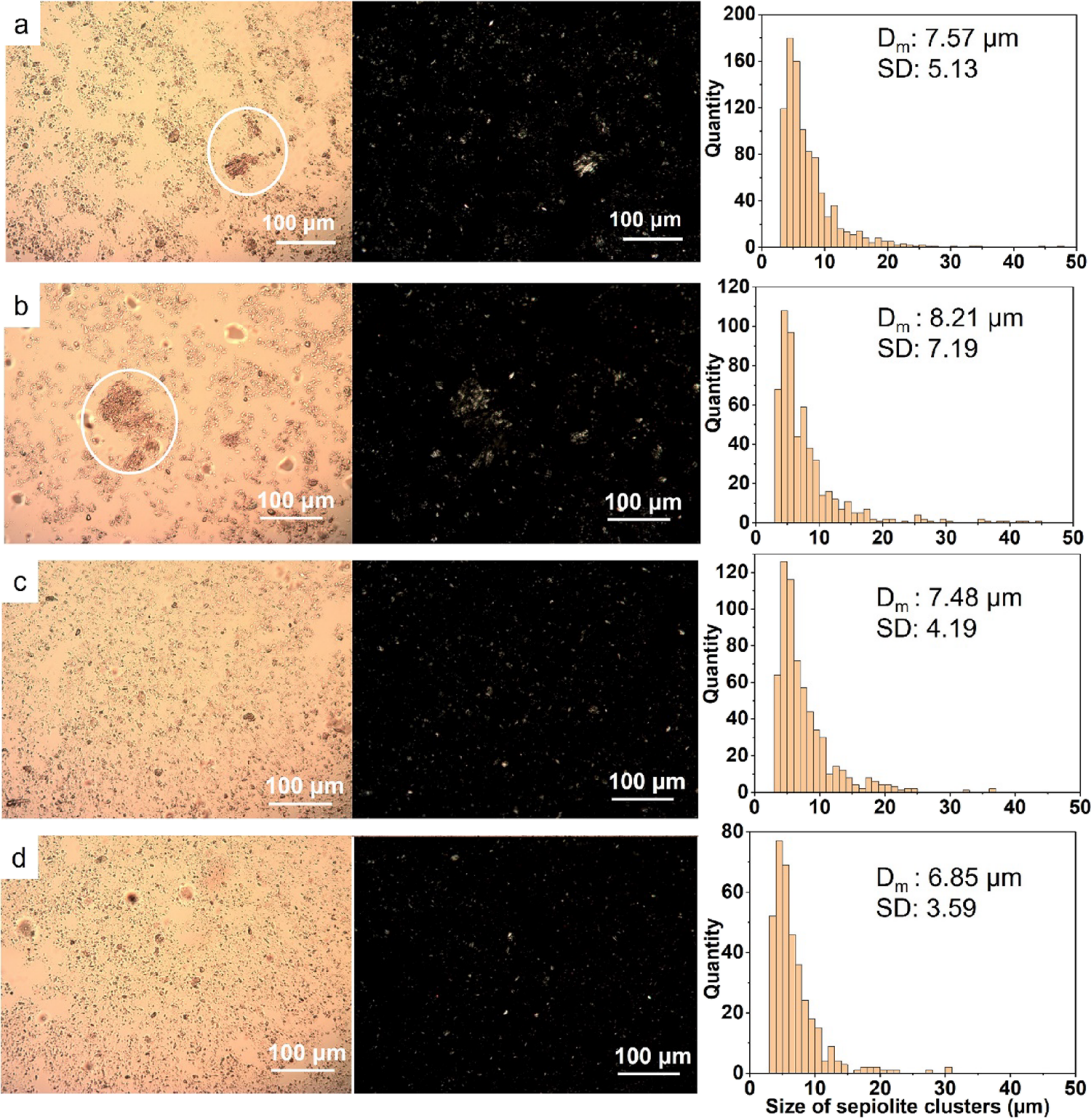
DIC microscopy (left), polarization (middle), and cluster size distribution (right) of the PAA/Sep mixture (with 1 wt % PAA and 2 wt % of sepiolite in DI water) using different mixing tools: (a) magnetic stirring at 600 rpm; (b) overhead blade mixer at 600 rpm; IKA UltraTurrax mixer at (c) 6000 rpm and at (d) 10,000 rpm. *D*_m_ is the mean diameter; SD is the standard deviation.

The dispersion of the nanoclays into the PAA solution was first investigated using the 1P2S (1% PAA and 2% sepiolite) group as an example. In Figure 2a,b, the mixtures were stirred by the magnetic bar and the blade mixer show clusters of sepiolite particles with a size of ∼100 and ∼50 μm, respectively. Those large clusters were largely absent when the UltraTurrax method was used (Figure 2c,d). With the use of the UltraTurrax at 10,000 rpm, the mean diameter of the sepiolite particles was only 6.85 ± 3.6 μm. It is thus evident that, of the mixing methods considered, the UltraTurrax mixer at a high mixing rate of 10,000 rpm appeared to achieve the most homogeneous dispersion of the clays, breaking up large aggregates.

#### 3.1.2 Effect of the Clay Distribution on the Surface Morphology of the PUF/1P2S Foam Samples

**Figure 3:**
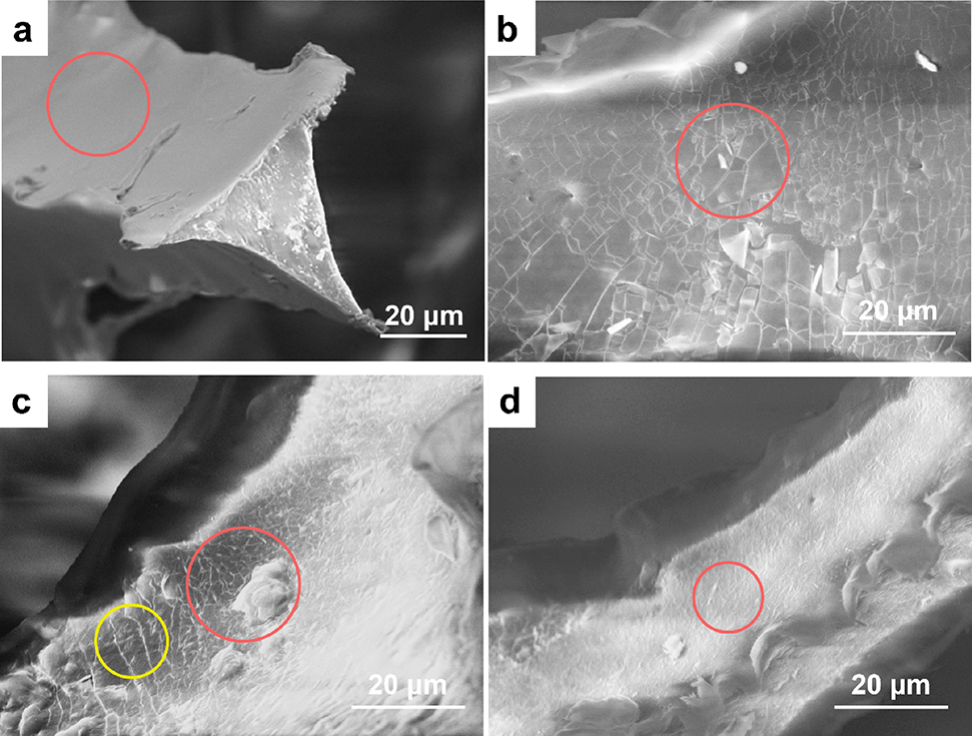
SEM images of (a) the pristine PUF strut, (b) PUF/1P, (c) PUF/1P2S (M), and (d) PUF/1P2S (UT10). The red circles highlight the surface features of the foam struts for comparison, and the yellow circle indicates a broken-glasslike surface in PUF/1P2S (M).

The mixing method affected the dispersion of the sepiolite particles in the PAA solution and, in turn, the surface morphology of the coated PUF samples. The red circles in Figure 3 highlight the same part of a foam strut in different samples. Compared to the smooth surface of the untreated PU foams (Figure 3a), the surface of PUF/1P (Figure 3b) showed a thin membrane of solidified PAA. This polymer membrane surface appeared brittle and fragmented, possibly due to the deformation of the foam struts when being cut for SEM observation. The morphology of the PAA/Sep coatings appears, however, significantly different. In Figure 3c, one can notice the presence of a cluster of clays with a diameter of ∼10 μm. Meanwhile, a brittle and fragmented coating was present on the surface of the foam (highlighted in the yellow circle), similar to the brittle PAA membrane in Figure 3b. Therefore, one can infer that the surface of the PUF/1P2S (M) coating consisted of a PAA region and a sepiolite reinforcement. It is assumed that PAA and sepiolite were detached from each other during the coating due to the poor dispersion provided by the magnetic stirring. However, in Figure 3d, a thin but dense sepiolite coating appeared to be present on the surface of the PUF/1P2S (UT10) sample without any obvious brittle membrane structure. The PAA and sepiolite have been homogenized well by the UltraTurrax mixer, providing a uniform coating onto the foam struts. This coating appeared to be sufficiently stable in terms of its surface structure during the coating procedure and after drying. Although a few small clay clusters can still be observed, it is evident that PUF/1P2S (UT10) was more homogeneously coated than the other foam samples.

#### 3.1.3 Effect of the Clay Distribution on the Mechanical Properties of the PUF/1P2S Foam Samples

**Figure 4:**
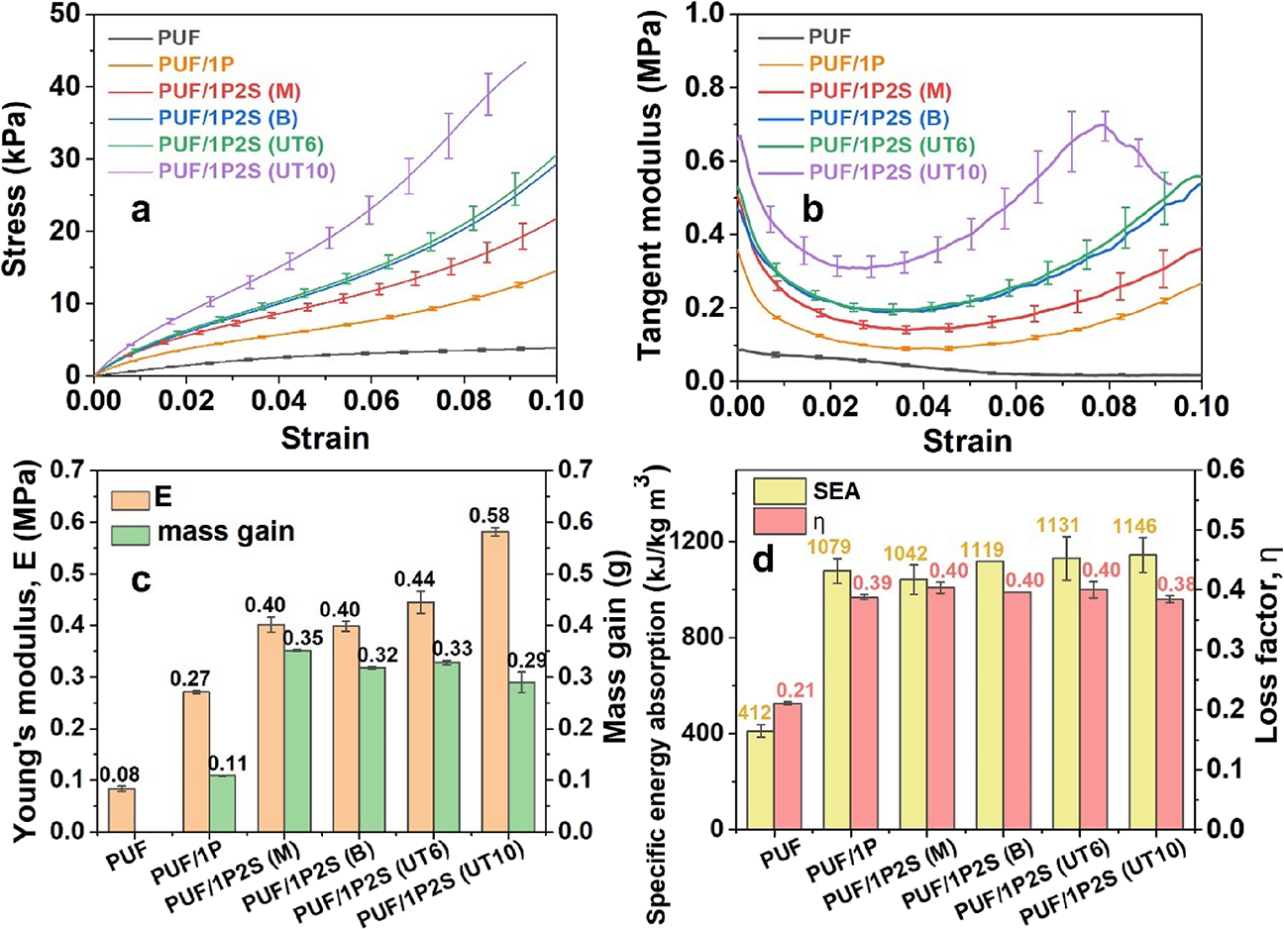
Quasi-static mechanical and energy dissipation properties of the PU foams coated with 1P and 1P2S. (a) Stress vs strain curves in compression; (b) tangent modulus; (c) energy absorption and weight gain; (d) specific energy absorption and loss factors.

The homogeneity of the distribution of nanofillers in a composite significantly affects its performance,^37,49−52^ especially in terms of mechanical properties. The PU foam samples made by different mixing methods have undergone a quasi-static compression test to investigate their properties. In Figure 4a–c, the foam samples that were most homogeneously coated (UT10) showed the largest stress and tangent modulus, with a Young’s modulus of 0.58 MPa. The other PUF/1P2S samples were however less stiff, with a Young’s modulus ∼0.4 kPa. This may be rationalized as follows. The PU skeleton of the PUF/1P2S (UT10) samples was strongly supported by the homogeneous dispersion of the PAA/Sep, resulting in an excellent mechanical performance. However, the use of other mixing techniques resulted in a poor distribution of the sepiolite within PAA (Figure 1), which created separate PAA and sepiolite aggregate regions on the foam struts (Figure 4c). Neither the PAA nor the sepiolite itself was sufficiently strong to protect the foam, as shown by the diminished stress and modulus. From an energy dissipation perspective, the PAA coating itself helped energy absorption (with the loss factor increasing from 0.21 to 0.39, Figure 4d), and the addition of 2 wt % sepiolite did not contribute to any significant increase in the loss factor (eq S1), no matter which mixing method was used. It is worth noticing that the average weight of PUF/1P2S (UT10) slightly decreased compared to the same coated samples made using the UT6, the magnet, or the blade mixers, possibly due to fewer clay aggregates being coated (Figure 3). This has also caused the highest SEA (specific energy absorption, eq S2) provided by the PUF/1P2S (UT10). The UltraTurrax mixer was therefore chosen with a rate of 10,000 rpm to take advantage of the uniform coating provided, with the aim of optimizing the overall mechanical properties of the PUF/PAASep composites in the subsequent work.

### 3.2 Mechanical Behavior of the PUF/1PnS Composites

#### 3.2.1 Quasi-Static Compression Tests

**Figure 5:**
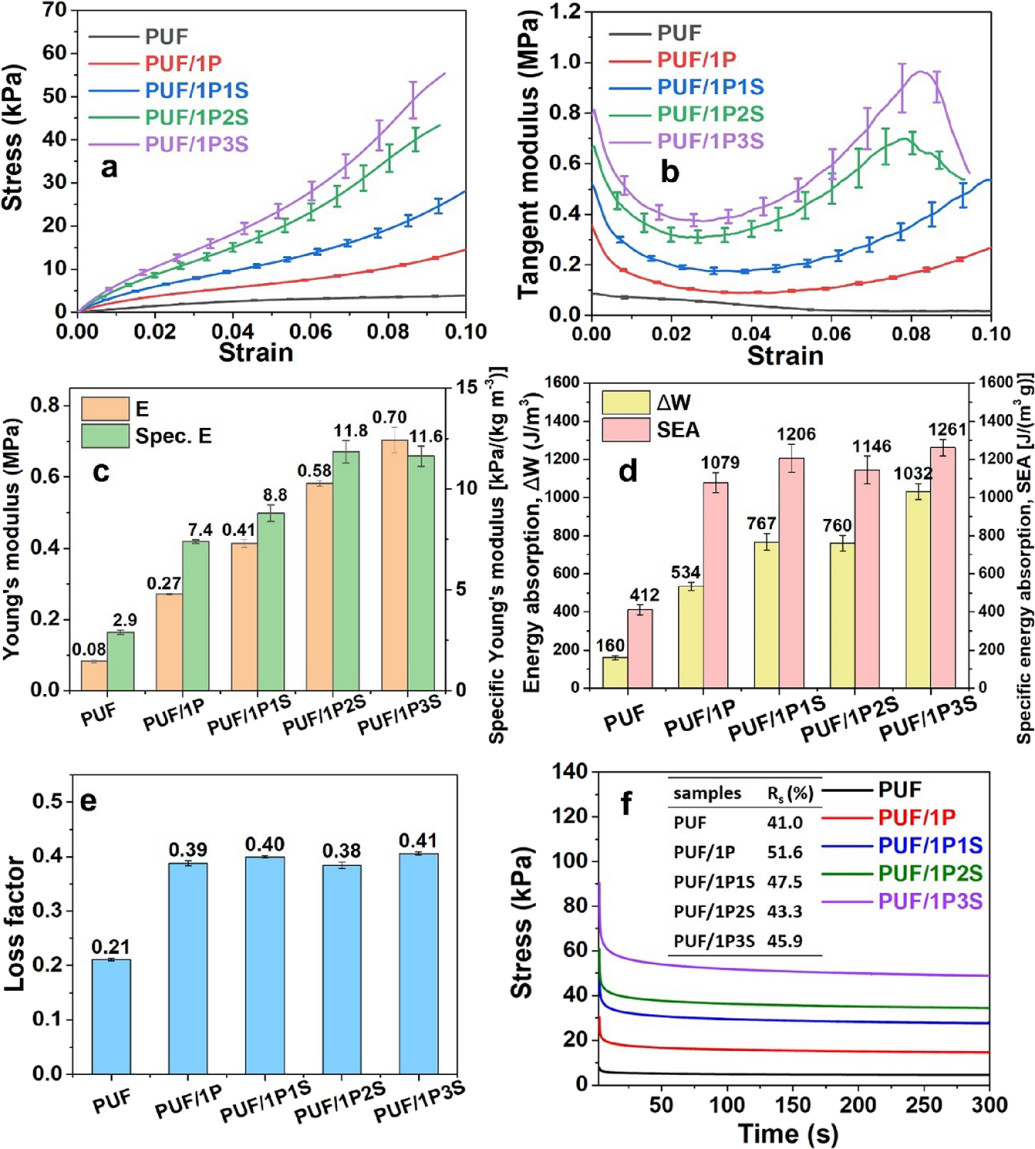
Quasi-static mechanical and energy dissipation properties of the PU foams coated with 1PnS using the UltraTurrax mixer at 10,000 rpm. (a) Stress vs strain curves, (b) tangent modulus, (c) Young’s modulus and specific Young’s modulus, (d) energy absorption Δ*W* and specific energy absorption (SEA), (e) loss factors, and (f) stress relaxation curves with the inserted table indicating the relaxation rate *R*_s_.

The PU foams coated with 1 wt % PAA and 1–3 wt % sepiolite were prepared and tested using cyclic compression-release loading. The neat PUF samples showed the lowest strength and stiffness (Figure 5a–c) with a Young’s modulus of 0.08 MPa. This value is similar to the flexible PU foam in other studies.^29,36,37^ The specific Young’s modulus was calculated as the ratio between the Young’s modulus and the sample density, to exclude the stiffening effect given by the density. The specific Young’s moduli of PUF/1P and PUF/1P2S were 7.4 and 11.8 kPa/(kg m^–3^), compared to the pristine foam (2.9 kPa/(kg m^–3^)). The behavior of the specific modulus indicates that the stiffening effect provided by the coating materials was significant for samples at equivalent weight. The 1 wt % PAA solution contributed to ∼28% of mass gain for each sample (Table 1), while the addition of more sepiolite increased the PUF/1P3S sample by ∼110% compared to the control untreated foams (mass ∼0.38 g), with a ∼80% mass gain from the sepiolite. The coated PAA and sepiolite particles improved the strength of the PU skeletons and contributed to the overall stiffness. The viscous PAA polymer can help to bond the sepiolite particles onto the foam surface and impede their detachment. Therefore, the PAA/Sep coating structures shown in Figure 2d provided a strong and solid protective coating for the foams, even at nanoscale.

The energy absorbed and the loss factors during the loading–unloading cycles are shown in Figures 5d,e, respectively. The PAA coatings enhanced the energy absorption capability with their intrinsic viscous nature, resulting in an approximately 3-fold increase in terms of Δ*W* and SEA and an increase by 0.18 in the loss factor compared with the uncoated samples. The mixture of PAA and sepiolite improved Δ*W* from 534 J m^–3^ for PUF/1P to 1032 J m^–3^ for PUF/1P3S, while the SEA was slightly increased by ∼190 J due to the addition of the sepiolite clays. Considering the scattering of the results, the loss factors could be, however, considered almost constant (∼0.40) with the addition of the sepiolite particles. The stiff sepiolite particles did not appear to contribute more to the energy absorption, whilst not diminishing the damping effect from PAA in quasi-static compressive tests.

Stress relaxation tests have been carried out to investigate the load bearing capability of the PU foam composites (Figure 5f). For each group of foams, the stress dropped dramatically when the loading force was removed and then gradually became stable. A significant relaxation effect could be observed when PAA was added, evident from the increase of the stress relaxation rate *R*_s_ (from 41% for PUF to 51.6% for PUF/1P; cf. eq S3). The *R*_s_ then decreased to 43.3% (PUF/1P2S) when stiff sepiolite particles were coated and slightly increased to 45.9% (PUF/1P3S), possibly due to the interfacial slippage between sepiolite particles. This increase is similar to the *R*_s_ observed from our previous work on sepiolite/chitosan lbl coatings.^36^

For comparison, PU foam samples coated with 1 wt % PAA and 4–5 wt % of sepiolite (PUF/1P4S and PUF/1P5S; cf. Table 1) have also been evaluated (Figure S2 in the Supporting Information). A substantial stiffening effect was provided by the sepiolite, resulting in a high stress and modulus. However, due to the large amount of sepiolite added to the PAA solution, it is likely that larger clusters of clays were present, resulting in nonuniform coatings. The inhomogeneity would lead to large differences in the modulus, absorbed energy, and loss factor between the samples. While the UltraTurrax mixer optimized the clay dispersion in PAA concentrations between 0–3 wt %, more powerful mixing techniques would be needed to prepare mixtures with higher clay concentrations.

#### 3.2.2 Dynamic Vibration and Transmissibility Tests

**Figure 6:**
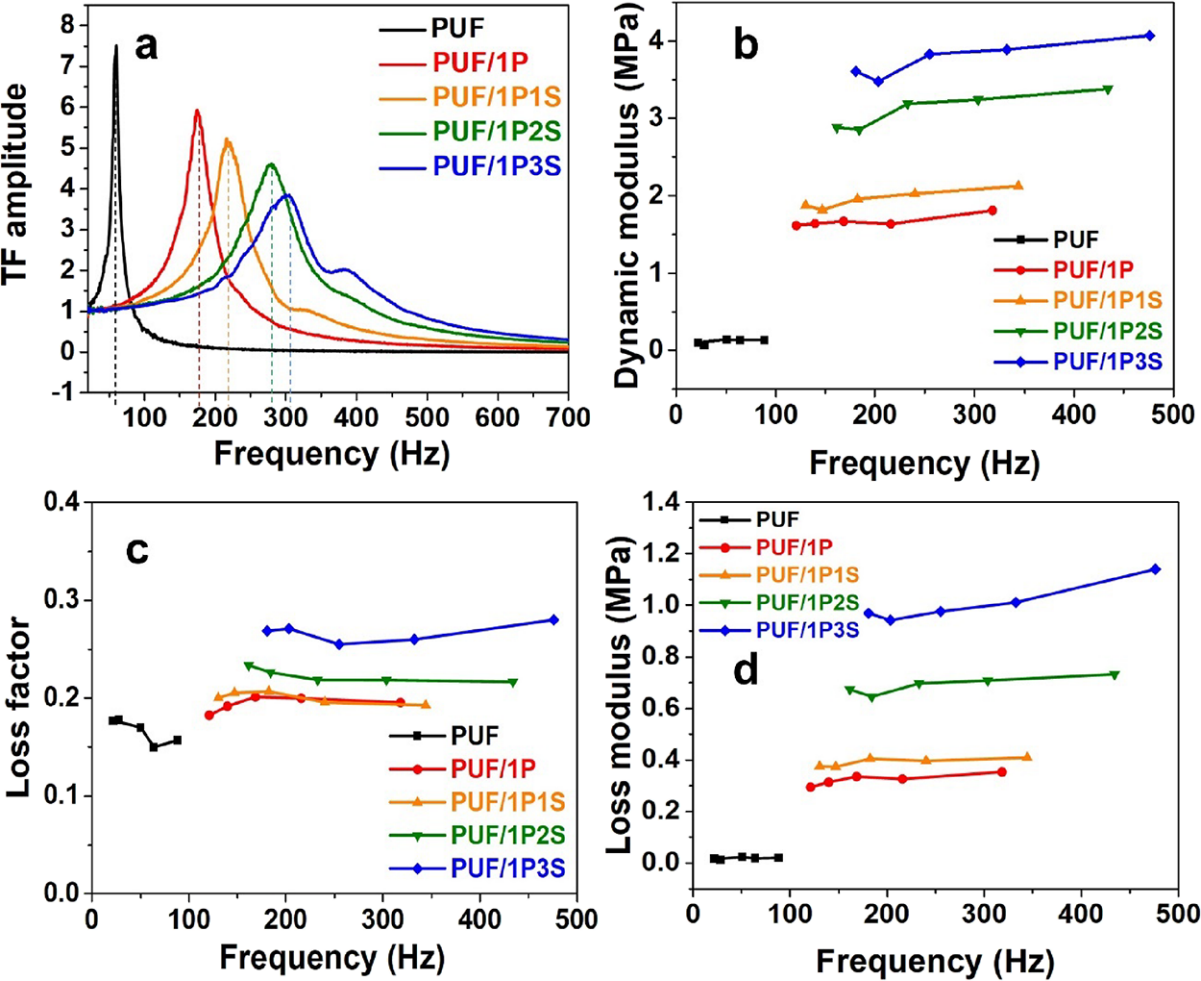
(a) Transfer functions, (b) storage modulus, (c) loss factor, and (d) loss modulus of the PUF samples coated with PAA and sepiolite at a base root-mean-square acceleration of 0.70*g*.

An effective attenuation of vibration is highly sought after in a wide range of engineering applications, such as automotive seatings, transportations, packaging, machining operations, and sound insulation, where flexible foams are used as cushions to dissipate energy. Figure 6a shows the transfer functions (TF) of the PUF samples coated with the PAA and sepiolite, tested at a base root-mean-square acceleration of 0.70*g* and a top mass of 64.0*g*. The uncoated PU foam exhibited a sharp and narrow response at a resonance frequency of ∼60 Hz. The PAA coatings contributed to an increase in the resonance frequency and a decrease in the peak amplitude at ∼180 Hz. Similarly, the sepiolite loading increased the resonance frequency to ∼220, 280, and 300 Hz, corresponding to an addition of 1, 2, and 3% sepiolite, respectively, while decreasing the peak TF amplitude. According to eqs S4 and S5, this means an improvement in both the storage modulus *E*_d_ and the loss factors η_d_ from the uncoated to the coated samples (Figure 6b,c).

In terms of *E*_d_, the pristine foam used in this study has a value of ∼0.13 MPa corresponding to the use of the smallest top masses, similar to *E*_d_ = 0.11 MPa for the PUF/chitosan/sepiolite system in our previous work.^36^ With large top masses, the *E*_d_ values were different (0.04 MPa in ref (36) and 0.07 MPa in this work). This may be caused by (1) the intrinsic anisotropy in the two different batches of raw foam materials and (2) the different plateau stresses from the larger deformation induced by the large top masses.

The stiffening effect is noticeable as the storage modulus increased with more coatings added to the PU foams. For each group of samples, five top masses were used to modify the resonance frequencies. One can observe an overall increase of the *E*_d_ for each sample because of smaller precompression and deformations caused by the small top masses (Figure 6b).^36^ It is also noticeable that the *E*_d_ values were always larger than the Young’s modulus from quasi-static tests for each group (Figure 5c). For the pristine foams, the value of *E*_d_ was ∼70% larger than the Young’s modulus *E*, while the *E*_d_ in the PUF/1P and PUF/1P3S samples was 5 times larger than their *E* values. This is because the great affinity between PU and PAA ensured a tightly binding PAA coating, resulting in a strong composite foam. In addition, the viscoelastic PAA material provided a stiffening effect of the foam under high strain rates, in addition to the pneumatic poroelastic force effect exerted onto the foams.^47^ The stiffening effect became more significant when sepiolite particles were added. For PUF/1P3S, the value of *E*_d_ was 4.07 MPa, which is 31 times that of *E*_d_ in the pristine PUF samples (0.13 MPa) with the same top mass.

The dynamic loss factor η_d_ of the samples is shown in Figure 6c. The pristine PU foam exhibited a range of loss factor within 0.14–0.18, similar to the η_d_ values in the literature.^14^ They were, however, much smaller than the coated PU foams. For the PUF/1P and PUF/1P1S samples, the loss factors were ∼0.20. However, PUF/1P2S showed an increase in the loss factor to 0.23, and this value increased to 0.28 for the PUF/1P3S samples. Unlike the decreased η_d_ due to the stiffened foam structures observed in PUF/sepiolite/chitosan composites,^36^ the foams coated with PAA and sepiolite showed a great energy dissipation capability. This can be explained as follows. Compared to the untreated foam, the PUF/1P dissipated more energy due to the viscosity of the PAA. With 1% sepiolite added, the coating layer became stiff and less susceptible to energy dissipation via deformation; however, the PAA/sepiolite coating could mediate local friction and slip during vibration. The friction may occur between (1) sepiolite particles, (2) sepiolite and PAA, and (3) between the PAA/sepiolite coating layer and the PU foams struts. During vibration, the energy can be partially dissipated via frictional effects, resulting in an equivalent η_d_ for PUF/1P1S and PUF/1P. For the PUF/1P2S and PUF/1P3S specimens, the high loading of sepiolite particles led to more local frictional effects; the thick PAA/clay coatings also decreased the equivalent pore size of the foams, which could also lead to an increase in both the modulus and energy dissipation.^53^ Therefore, the energy was efficiently dissipated in the PUF/1P2S and PUF/1P3S samples. The measured dynamic loss factors, for example, 0.15 in PUF and 0.28 in PUF/1P3S, are smaller than those obtained from the quasi-static tests (0.21 in PUF and 0.40 in PUF/1P3S). This is mainly because the maximum strain in the cyclic quasi-static test was 0.1, compared with the much smaller (lower than 0.02) strain in the transmissibility tests.^47^ The larger deformation would cause more significant local deformation and micro friction of the coating, leading to larger loss factors in the quasi-static tests.

In Figure 6d, the loss modulus *E*_l_ (the product between the storage modulus and loss factor; eq S6) is indicative of the overall damping performance from the vibration transmissibility tests. Because of the larger *E*_d_ and η_d_ values of the PUF/1P3S sample, it showed a remarkable *E*_l_ of 1.14 MPa at 476 Hz. The PUF/1P2S exhibited the second largest *E*_l_ value of 0.73 MPa at 434 Hz. The *E*_l_ values of the PUF/1P1S and the PUF/1P were around 0.4 MPa, in contrast to 0.02 MPa for the uncoated foam samples. The PUF/1P3S sample benefited from the significant stiffening and energy dissipation due to the PAA/sepiolite coatings, resulting in a *E*_l_ that is 57 times that of the untreated PUF samples.

The vibrational amplitude has also been modified by adjusting the base acceleration rate (Figure S3). The PUF composites showed a slight nonlinear softening response, as the higher base amplitude led to a decrease in both the resonance frequency and the peak TF values. This is because the intense vibration could trigger larger deformation with slightly decreasing tangent modulus (Figure 5b), which can lead to a decrease of *E*_d_, as well as an increase of η_d_. The softening phenomenon at small strain range is mainly caused by the microcracks of the coating and the nonlinear deformation of the microstructures inside the porous materials. More microcracks could be produced in the thick and brittle coating layer, so the nonlinearity appears significant for the PUF/1P3S group.

#### 3.2.3 Impact Properties

**Figure 7:**
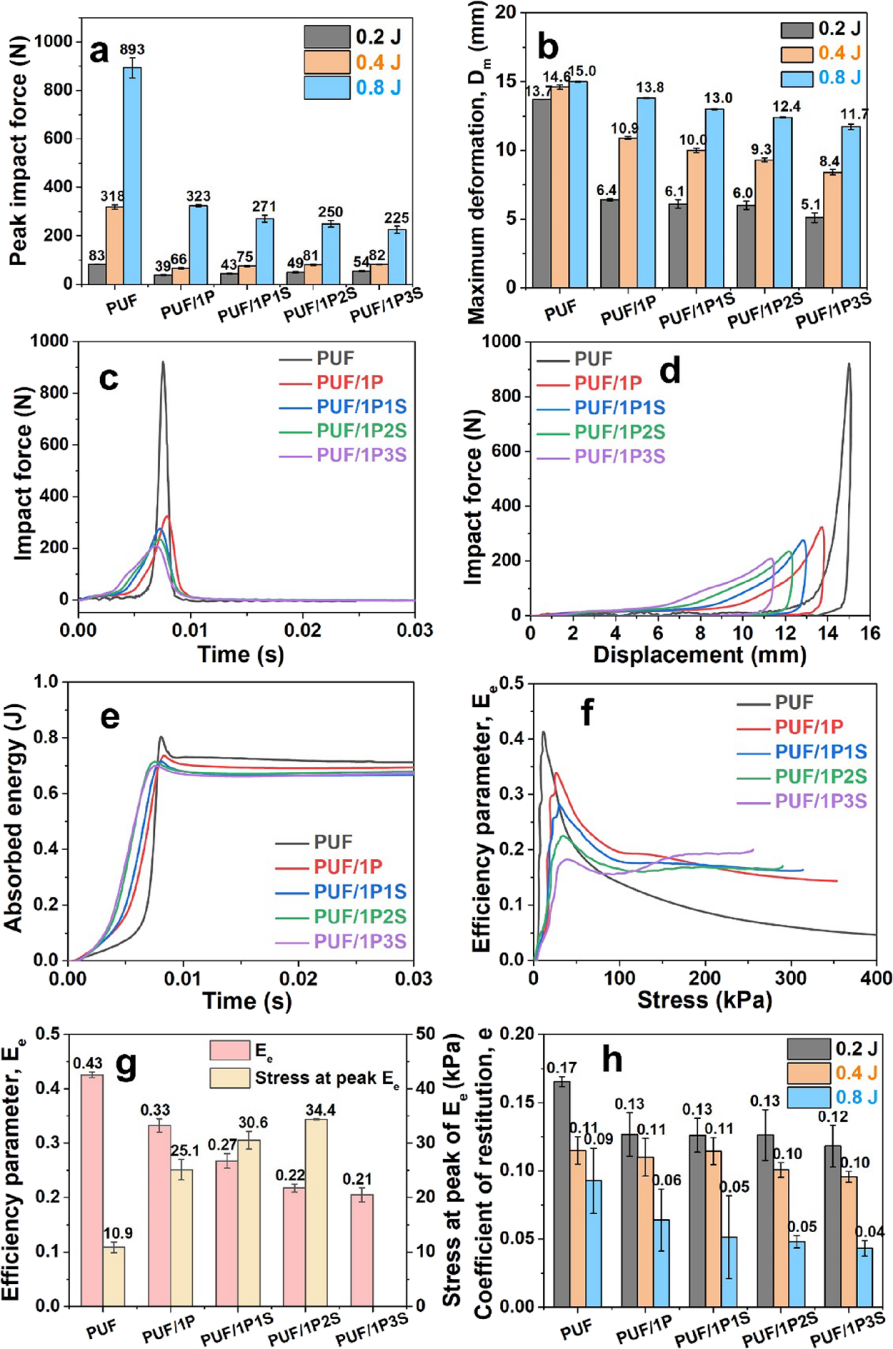
Impact performance of the PU foam samples coated with PAA and sepiolite. (a) Peak impact force values; (b) maximum deformation of the samples with the three groups of impact energy. Data in panels (c–g) are related to the 0.8 J energy group. (c) Time history of the force, (d) force–displacement curves, (e) time history of the absorbed energy, (f) efficiency parameter *E*_e_ vs stress, and (g) peak *E*_e_ value and the stress at the peak *E*_e_ for each sample group. (h) Coefficient of restitution *e* of the samples with three energy groups.

As a cushioning material, PU foams are required to provide satisfactory resistance to the external impact force.^54^ The impact properties described in this work have been evaluated using three energy values of 0.2, 0.4, and 0.8 J, achieved by adjusting the height of the drop mass. The peak impact force that transmitted through the samples is shown in Figure 7a, with the maximum deformation in Figure 7b. When impacted, the uncoated foams transmitted most force and were deformed more significantly; the coated foams, however, showed a smaller impact force with smaller sample deformations. For example, the peak impact force noticeably decreased from 893 N (PUF) to 323 N (PUF/1P), then further decreased to 225 N (PUF/1P3S) in the 0.8 J group (Figure 7a). In the 0.2 and 0.4 J group, the deformation of the coated foams was much smaller than that of the pristine PUFs, because the impact energies were not large enough to initiate the impact resistance of the whole foam sample. For the 0.8 J group, the impact force that exerted onto the pristine PU foam was not well dissipated, as demonstrated by the sharp and narrow force history curve (Figure 7c) and a concave force–displacement loop (Figure 7d). Overall, the PU foam composites showed an obvious force resistance and longer impact duration with smaller force–displacement loops. This is because the pristine PU foams are soft and flexible, and their cell struts are easily bent and buckled by the drop mass, leading to large deformations. The coatings strengthen the PU struts, especially in the case of the PAA/sepiolite coatings. This strengthening prevents the foam skeleton from being seriously deformed. Furthermore, the foam struts with thick coating layers appear to reach densification at lower displacements, resulting in a greater impact resistance at small deformations.

The absorbed energy was calculated using eq S7 (Figure 7e). The impact energy increased fast during the impact for each sample group and then decreased to a constant value because of the rebound. The uncoated PU foam showed a largest *W* peak value among all the foam samples due to its large deformation, followed by a rapid decrease as the absorbed energy was quickly transformed into the rebound energy. On the other hand, the final *W* value of the coated foam samples was slightly smaller than the one of the uncoated counterpart. Overall, this could be attributed to the friction effects of the coating layer, which help to dissipate energy. Another factor affecting the energy absorption performance is the small impact deformation of the coated foams that reduces the dissipated energy.

The efficiency parameters *E*_e_ are calculated as a function of the impact stress (eq S8, Figure 7f). The *E*_e_ value of the pristine PU foam was 0.43 at 10.9 kPa, while it dropped to 0.2 at ∼50 kPa. It further decreased and went well below 0.1 at ∼200 kPa. These data are comparable to open cell PU and other EPS types of foams evaluated in the open literature.^36,48,54^ For the coated foams, the efficiencies showed maximum values within 0.21–0.33, while the *E*_e_ curves tapered down more slowly than in the case of the pristine foam. All PAA/sepiolite-coated foams showed *E*_e_ values above 0.15 from 100 kPa onward. Each sample group showed a peak *E*_e_ at a specific stress, except for PUF/1P3S, which showed an increasing efficiency when the stress went beyond 150 kPa. In Figure 7g, the stress values at peak *E*_e_ are 10.9, 25.1, 30.6, and 34.4 kPa corresponding to the PUF, PUF/1P, PUF/1P1S, and PUF/1P2S samples, respectively. It is evident that the coated PU foams were efficient within a wider and larger range of stresses, especially when they were used under a high stress.

The coefficient of restitution *e* was calculated using eq S9 to compare the kinetic energy of the drop mass before and after impact. Figure 7h shows the coefficient *e* over the three energy groups. A higher impact energy caused larger deformations that help to dissipate more kinetic energy, leading to lower *e* values. Within the same energy group, the coated foams tended to dissipate an increasing proportion of kinetic energy, causing a decrease in the coefficients of restitution. Considering the scatter of the results, there was no obvious difference between the PAA-coated foams and the PAA/Sep-coated ones. Nevertheless, the PAA/Sep-coated PUFs show great potential to resist impact forces and to act as useful cushion foams.

#### 3.2.4 Energy Dissipation Mechanisms

**Figure 8:**
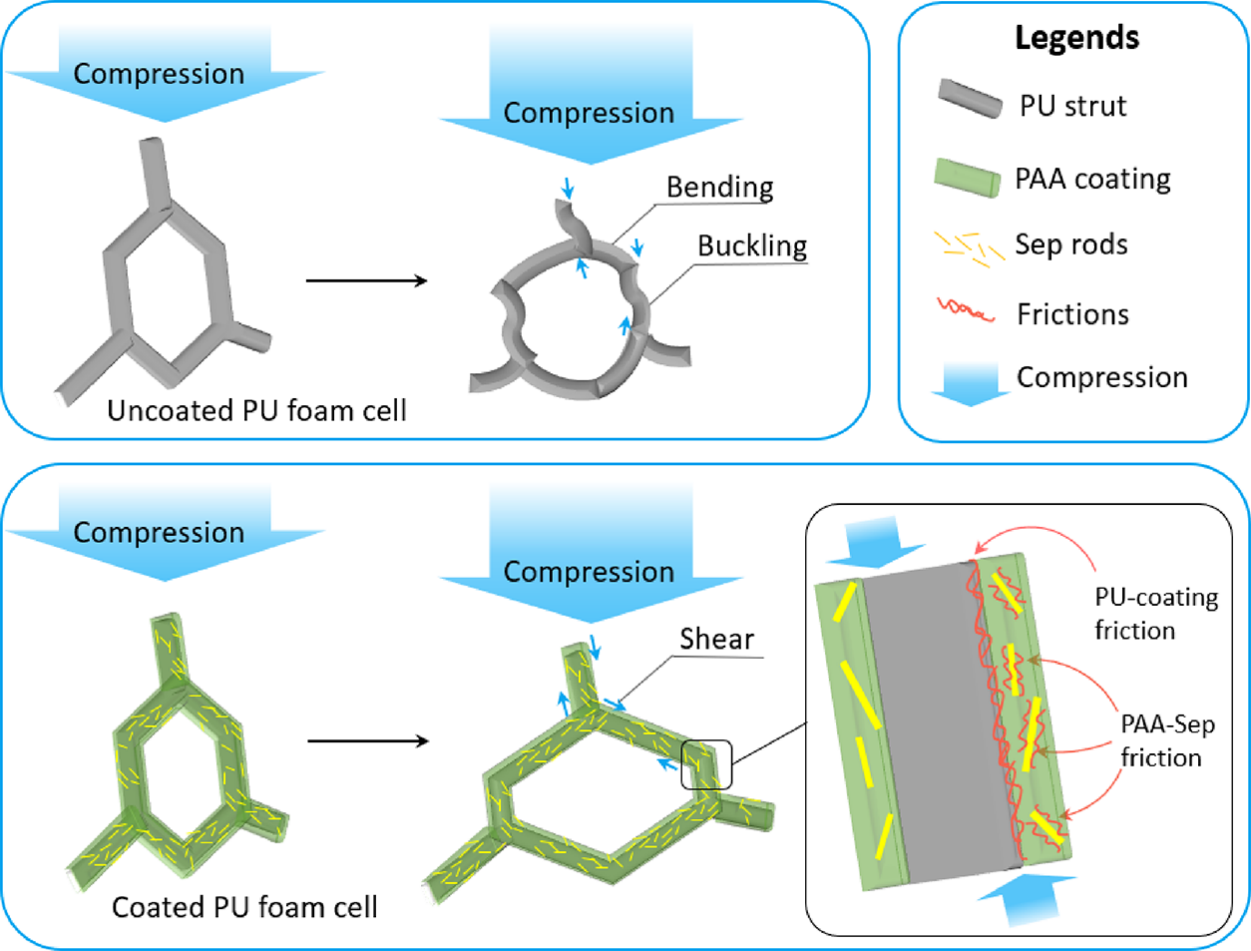
Schematic of the damping mechanisms in the PU foam samples before and after the application of PAA/Sep coatings.

Figure 8 describes the energy damping mechanism in the untreated PUF and PUF/PAA/Sep systems. At the microscopic level, the PU foams are made of cells with flexible PU struts. The struts have coaxial PAA/Sep coatings in which sepiolite nanorods distribute evenly in the PAA matrix by using the mixing tools. When an external mechanical compressive/tensile loading is exerted, the struts of the uncoated foams are flexible and deform by bending and buckling.^29,55^ However, the intrinsically hard PAA/Sep coatings reinforce the foam by providing a strong protective layer that prevents the struts from being bent, with the resulting deformation dominated by axial compression/stretching and shear. Those compression/shear deformations activate slip/stick and interface friction mechanisms with the PU and PAA/Sep coatings that dissipate energy. As a result, PUF/PAASep composite systems achieve both high stress and damping factors.

#### 3.2.5 Comparison of the Mechanical Properties with Previous Studies

**Figure 9:**
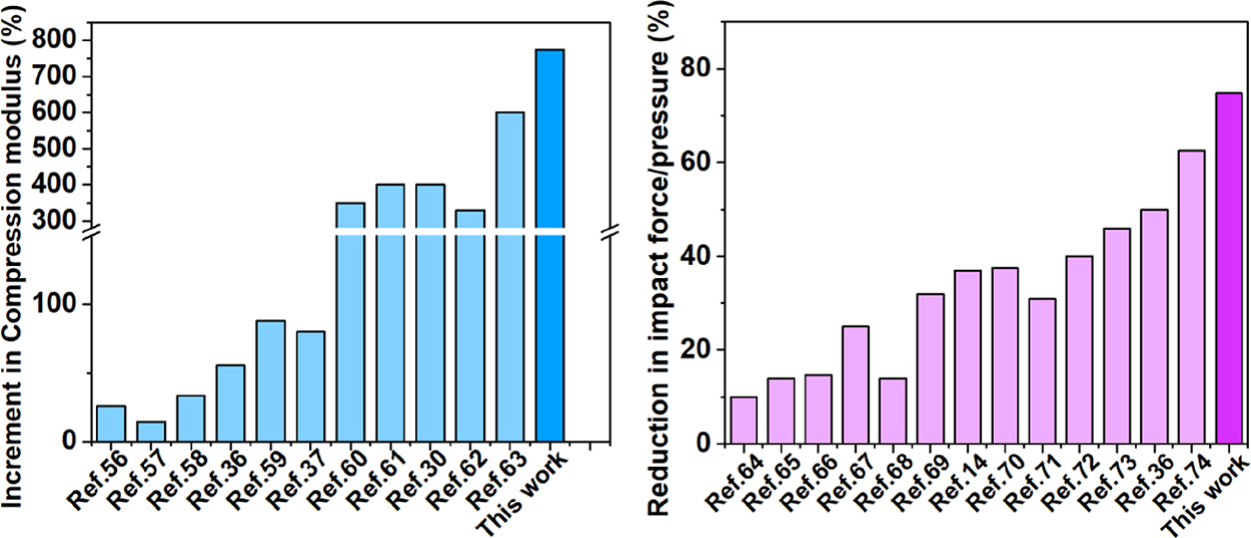
Increase in (a) Young’s or compressive modulus and (b) impact force reduction from other polyurethane foam composites described in the open literature.

In Figure 9a, we compared the increment of the Young’s or compression modulus on polyurethane foam composites.^30,36,37,56−63^ For example, Cura et al.^30^ have modified PU foams with carbon nanotubes and polyurethane dispersions, achieving a 400% increase in compression modulus. The impact resistance was compared with that of other polyurethane foam composite cushions in the literature^14,36,64−74^ (Figure 9b). For example, Yang et al.^70^ prepared aerogel-incorporated PU foams, resulting in a 37.5% decrease in terms of the peak impact force. Glass fiber reinforced PU foam composites designed by Yu et al.^67^ reduced the impact pressure by 25% compared to that of untreated foams. The simple and cost-effective preparation of PUF/PAA/Sep composites proposed in our work achieved a very large and significant improvement in terms of compressive modulus and impact resistance compared to other solutions proposed in the literature.

## 4 Conclusions

In this work, a PAA/sepiolite composite coating was designed and coated onto flexible polyurethane foams. The SEM morphology showed a uniform and homogeneous coating present on the surface of the foam struts with nanocoatings processed using an IKA UltraTurrax mixer at 10,000 rpm. This resulted in an excellent mechanical performance compared to other PU foam composites described in previous studies. Coated with 1P3S, the stiffened PU foams showed Young’s modulus 8 times that of the untreated foams, and the impact force was decreased by the factor of 3 in the large impact energy group. The PAA/sepiolite coatings produced micro frictions and coating–foam interactions during energy absorption; thus, the SEA of PUF/1P3S was three times that of uncoated PUFs. Particularly, the loss modulus of 1P3S was 57 times that of the pristine foams, as an overall consequence of the stiffening effect and the energy damping ability provided by the PAA/sepiolite coatings. Our findings thus demonstrate that the coated PU foams have great potential to be used in applications such as cushioning, supporting, and protective materials.

## Supporting Information Available

Equations for the mechanical parameters; quasi-static test results for PUF/PAASep samples at other formulations; transmissibility test results for PUF/PAASep samples at different base accelerations (PDF)
