# Supplementary material for: Giant Cushioning Effect in Facile Polymer/Nanoclay-Coated Flexible Polyurethane Foams
Source: ACS Appl Polym Mater. 2024 Aug 22;6(17):10322–33. doi: 10.1021/acsapm.4c01437 (PMC11406485; doi:10.1021/acsapm.4c01437)
Supplement: Supplementary file 1 — ap4c01437_si_001.pdf [file ap4c01437_si_001.pdf]

Supporting Information

Giant cushioning effect in facile polymer/nanoclay coated flexible polyurethane foams

Wenfei Ji[1], Qicheng Zhang[2], Jeroen S. van Duijneveldt[1], Wuge H. Briscoe[1,], Fabrizio Scarpa[2,*]*

[1]School of Chemistry, University of Bristol, Cantock's Close, Bristol BS8 1TS, UK.

[2]Bristol Composites Institute, University of Bristol, University Walk, Bristol BS8 1TR, UK.

[*]Email: wuge.briscoe@bristol.ac.uk; f.scarpa@bristol.ac.uk

1. Equations for the mechanical parameters

$$\eta_c = \frac{2\Delta W}{\pi U} \tag{S1}$$

$$SEA = \frac{e}{m} \tag{S2}$$

$$R_s = 1 - \frac{S_{final}}{S_{peak}} \tag{S3}$$

$$E_d = \frac{{\omega_n}^2 h}{A}(m_1 + \frac{m_2}{3}) \tag{S4}$$

$$\eta_d = \frac{1}{\sqrt{\beta^2 - 1}} \tag{S5}$$

$$E_l = E_d \eta_d \tag{S6}$$

$$W = \int_0^{\varepsilon} \sigma(\varepsilon) d\varepsilon \tag{S7}$$

$$E_e = \frac{\int_0^{\varepsilon} \sigma(\varepsilon) d\varepsilon}{\sigma(\varepsilon)} \tag{S8}$$

$$e = \frac{|V_{af}|}{|V_{bf}|} = \sqrt{\frac{E_{bf} - W}{E_{bf}}} \tag{S9}$$

η_c and η_d are the loss factor in the quasi-static tests and the dynamic tests, respectively. ρ is the density, *m* is the mass of the sample, *V* is the volume.

For the compression tests, ΔW is the energy dissipated in the hysteresis loop, *U* is the area under the middle curve of the hysteresis loop. *E* refers to Young's modulus. *SEA* refers to the specific energy absorption. *e* is the energy absorbed during compression. R_s is the stress relaxation rate, S_{peak} and S_{final} is the maximum and final stress in the relaxation tests.

For the transmissibility tests, m_1 is the given top mass, m_2 the mass of the sample, *A* the surface area, and *h* the height of the sample. The peak value of the TF amplitude is given as β with the corresponding frequency marked as ω_n.

For the impact property tests, the absorbed impact energy W and the efficiency parameter are calculated using the stress σ and strain ε. The coefficient of restitution *e* is a comparison between the velocity of the impactor before (V_{bf}) and after (V_{af}) impact. E_{bf} is the kinetic energy before

the impact.

2. Quasi-static compressive properties for PUF/PAASep composites at other formulations

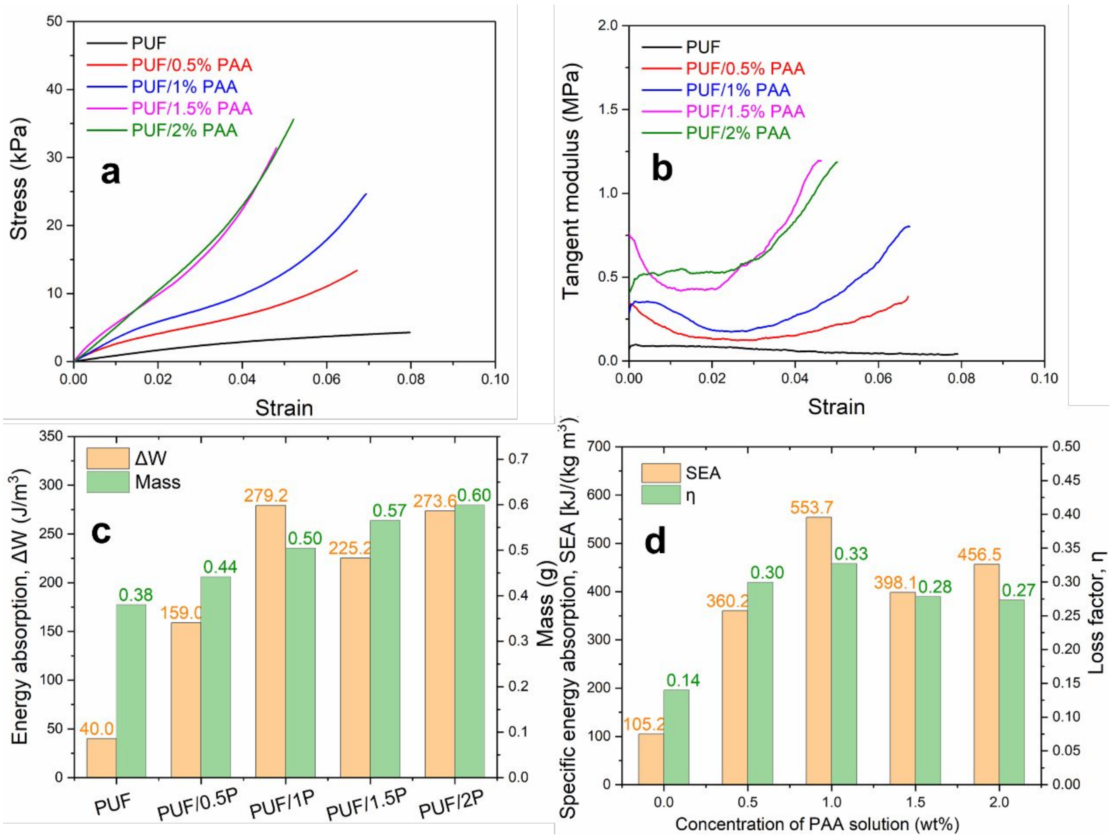

Figure S1. Quasi-static mechanical and energy dissipation properties of the PU foams coated with different concentrations of PAA. The stress vs strain curves (a); tangent modulus (b); the energy absorption and the weight gain (c); the specific energy absorption and the loss factors (d).

An increasing stress and modulus were shown with 0-1.5% of PAA and kept constant when the PAA concentration went up from 1.5% to 2 %. However, the SEA and the loss factor increased from 0 to 1% PAA, then reduced from 1% to 2% PAA. Therefore, 1% PAA was used to optimise the energy dissipation capability of the PAA coatings.

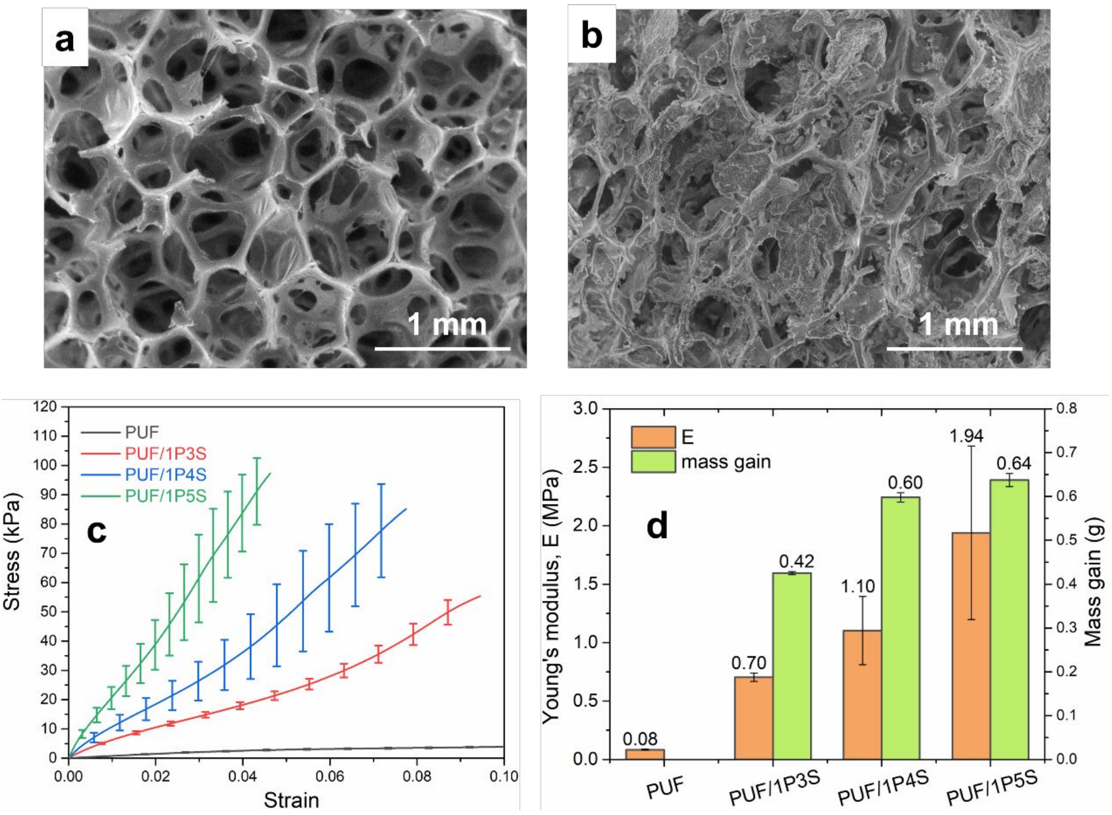

Figure S2. The morphology of (a) the PUF foam, (b) the PUF/1P5S sample. The results from the quasi-static tests: (c) the stress vs strain curves, (d) the Young's modulus and the mass gain for each sample group.

3. Transmissibility tests for the PUF/PAASep samples with different base accelerations

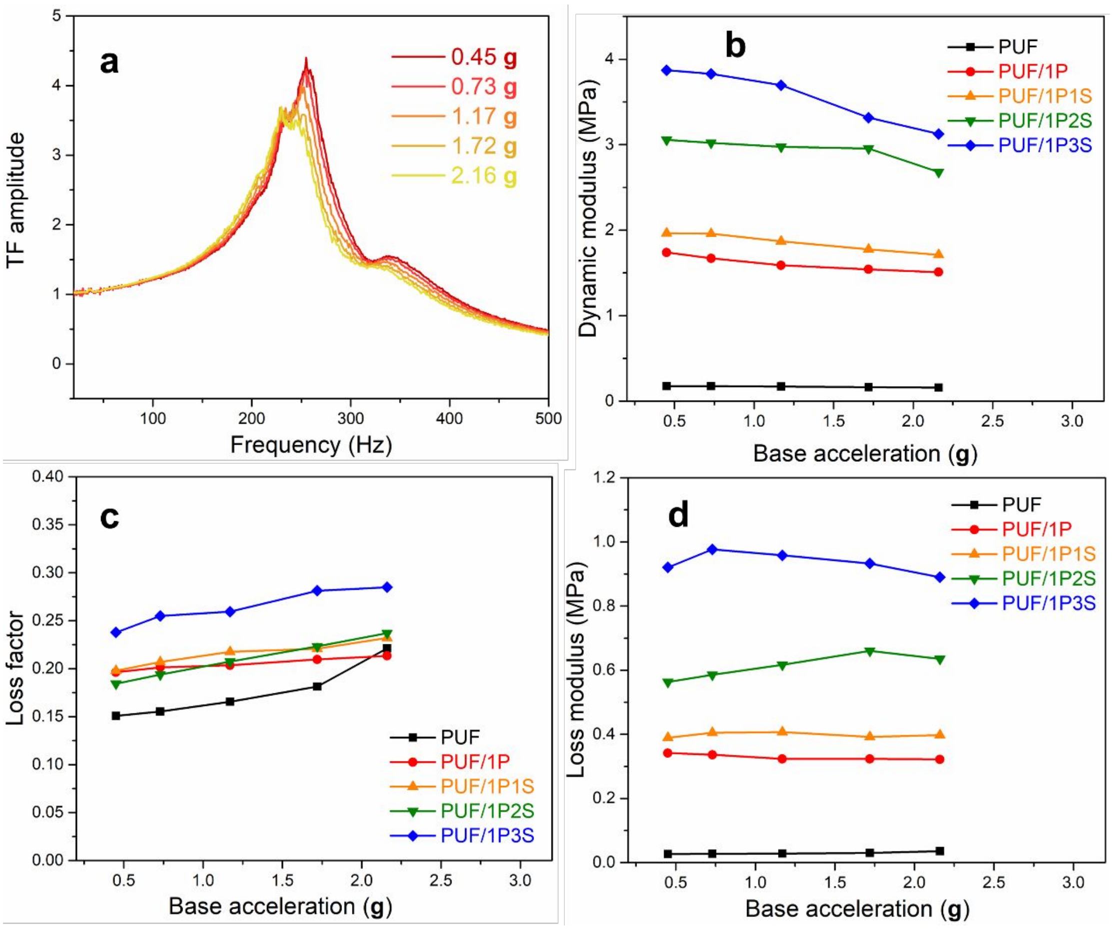

Figure S3. The transfer function for the PUF/1P2S sample tested with different base accelerations (a), the dynamic modulus (b), loss factors (c) and the loss modulus (d).
